# Opposing roles of Fos, Raw, and SARM1 in the regulation of axonal degeneration and synaptic structure

**DOI:** 10.3389/fncel.2023.1283995

**Published:** 2023-11-30

**Authors:** Thomas J. Waller, Catherine A. Collins

**Affiliations:** ^1^Department of Molecular, Cellular, and Developmental Biology, University of Michigan, Ann Arbor, MI, United States; ^2^Department of Neurosciences, Case Western Reserve University, Cleveland, OH, United States

**Keywords:** Wallerian degeneration, *Drosophila* NMJ, JNK, ASK1, synaptic growth, bouton structure, delayed degeneration

## Abstract

**Introduction:**

The degeneration of injured axons is driven by conserved molecules, including the sterile armadillo TIR domain-containing protein SARM1, the cJun N-terminal kinase JNK, and regulators of these proteins. These molecules are also implicated in the regulation of synapse development though the mechanistic relationship of their functions in degeneration vs. development is poorly understood.

**Results and discussion:**

Here, we uncover disparate functional relationships between SARM1 and the transmembrane protein Raw in the regulation of Wallerian degeneration and synaptic growth in motoneurons of *Drosophila melanogaster*. Our genetic data suggest that Raw antagonizes the downstream output MAP kinase signaling mediated by *Drosophila* SARM1 (dSarm). This relationship is revealed by dramatic synaptic overgrowth phenotypes at the larval neuromuscular junction when motoneurons are depleted for Raw or overexpress dSarm. While Raw antagonizes the downstream output of dSarm to regulate synaptic growth, it shows an opposite functional relationship with dSarm for axonal degeneration. Loss of Raw leads to decreased levels of dSarm in axons and delayed axonal degeneration that is rescued by overexpression of dSarm, supporting a model that Raw promotes the activation of dSarm in axons. However, inhibiting Fos also decreases dSarm levels in axons but has the opposite outcome of enabling Wallerian degeneration. The combined genetic data suggest that Raw, dSarm, and Fos influence each other's functions through multiple points of regulation to control the structure of synaptic terminals and the resilience of axons to degeneration.

## Introduction

Axons are extensive, vulnerable components of neuronal circuits. When an axon is severed from its cell body, it undergoes a self-destruction process known as Wallerian degeneration (Waller, 1850), akin to apoptosis, but invoking distinct molecular machinery. Exciting discoveries over the past 15 years have revealed key elements of this machinery (Gerdts et al., 2016; Coleman and Höke, 2020), whose biochemical functions have been linked to the synthesis and breakdown of the electron carrier nicotinamide adenine dinucleotide (NAD+). SARM1 (sterile alpha and TIR motif containing 1) is a key enzyme whose TIR domains catalyze the breakdown of NAD+. Unmitigated activation of these TIR domains leads to NAD+ loss and metabolic catastrophe (Gerdts et al., 2015). A potent negative regulator of SARM1 is the NAD+ biosynthetic enzyme NMNAT (Figley et al., 2021). NMNAT levels are tightly regulated in axons, and the loss of NMNAT leads to the activation of SARM1′s NADase activity, which then drives axonal degeneration (Gilley and Coleman, 2010; Gerdts et al., 2015; Gilley et al., 2015).

In contrast to the enzymatic activities of SARM1 and NMNAT, several studies have suggested that axonal degeneration may also be influenced by transcriptional mechanisms (Xiong and Collins, 2012; Farley et al., 2018; Hao et al., 2019). In this case, gene expression programs may influence the composition of axons making them more or less resilient to degeneration. Transcriptional mechanisms are not expected to function in acutely injured axons since severed axons are disconnected from the nucleus but are expected to be important for adaptive responses to chronic stressors, such as the presence of chemotherapeutic agents, neuropathies, or neurodegenerative disease.

Our previous study discovered a transcriptional pathway in *Drosophila* neurons that delays the rate at which injured axons degenerate (Hao et al., 2019). This pathway is restrained by the transmembrane protein Raw and is dependent on the Fos transcription factor. The mechanism by which this pathway protects axons is not known but appears to be independent of the levels of NMNAT (Hao et al., 2019); this contrasts the mechanism of Raw with other known regulators of axonal degeneration (Xiong et al., 2012; Walker et al., 2017).

Here, we probe the mechanism of Raw with respect to the SARM1 enzyme. For this, it is important to consider that in addition to its highly studied function in driving axonal degeneration as a NADase enzyme, SARM1 also functions as a regulator of MAP kinase signaling (Waller and Collins, 2022). SARM1′s ortholog in *C. elegans*, TIR-1, functions as part of a MAP kinase signaling pathway that controls a cell fate choice, neural communication, and innate immunity (Couillault et al., 2004; Chuang and Bargmann, 2005; Inoue et al., 2013). Similarly, in *Drosophila* and mammalian nervous systems, SARM1′s orthologs have been shown to function within MAP kinase signaling cascades that control presynaptic structure (McLaughlin et al., 2016), glial phagocytosis (McLaughlin et al., 2019), and cytokine expression (Wang et al., 2018).

Raw is known to antagonize JNK signaling in *Drosophila*, and its *C. elegans* ortholog OLRN-1 antagonizes the MAP kinase pathway engaged by TIR1 (Bauer Huang et al., 2007). A key mediator of this pathway is the MAPKKK ASK1, which is also engaged by dSarm in *Drosophila* (Brace et al., 2022; Herrmann et al., 2022). We therefore hypothesized that Raw functionally intersects with dSarm to regulate axonal degeneration. Here, we studied the relationship of Raw with dSarm in the regulation of axonal degeneration and nuclear signaling downstream of dSarm. We found that loss of Raw and overexpression of dSarm cause similar synaptic overgrowth phenotypes that are dependent on the Fos transcription factor, suggesting that Raw restrains a nuclear pathway engaged by dSarm. However, Raw shows a different relationship with dSarm in the regulation of axonal degeneration. Our genetic data suggest that Raw promotes dSarm function in degenerating axons while antagonizing dSarm-induced signaling to regulate synaptic growth. We propose that Raw and dSarm functionally intersect at multiple junctures to control the resilience and growth of axons and synaptic terminals.

## Materials and methods

### Fly stocks

The following fly lines were used: W118 (WT), UAS-Luciferase (Control Protein) (BDSC 35788), UAS-*lexA*-RNAi (Control RNAi 1) (BDSC 67947), UAS-*luciferase*-RNAi (Control RNAi 2) (BDSC 31603), QUAS-gRNA (Control gRNA) (BDSC 67539), UAS-Homo-TIR (gift from DiAntonio lab), UAS-GFP-Raw (Lee et al., 2015), UAS-*raw*-RNAi (VDRC 101255), *raw*^*dcp*−1^ (Hao et al., 2019), *raw*^134.47^ (Jemc et al., 2012), UAS-GFP-Raw (Lee et al., 2015), UAS-dNMNAT (Zhai et al., 2006), UAS-dSarm (BDSC 17144), UAS-dSarm::GFP (II and III) (Osterloh et al., 2012). UAS-Fos^DN^ (II) [BDSC 7214, (Eresh et al., 1997)], UAS-Fos^DN^ (III) [BDSC 7215, (Eresh et al., 1997)], UAS-Bsk^DN^ (BDSC 9311), UAS-Bsk-HA (F003890), *puc*-LacZ (Martín-Blanco et al., 1998), dSarmΔTIR [known as dSarm-ARM-SAM in (Herrmann et al., 2022)], BG380-Gal4 (Budnik et al., 1996), D42-Gal4 (Sanyal, 2009), M12-Gal4 (Ritzenthaler et al., 2000), UAS-Dcr2 (BDSC 24650), and UAS-Cas9 (BDSC 58985). dSarm-3x-guide RNA was provided by Dion Dickman.

### Dominant-negative constructs

The Fos^DN^ fly line (Eresh et al., 1997) (contains the bZIP (basic Leucine Zipper) domain of Fos (Kay) required for DNA binding and dimerization with other transcription factors (including Jun and TATA box-binding protein) (Ransone et al., 1993) but missing the rest of the protein required for activating transcription (Lloyd et al., 1991). The Bsk^DN^ transgene contains the K53R mutation in the ATP binding site of Bsk, which is shown to inhibit JNK signaling in *Drosophila* (Weber et al., 2000).

### dSarm-3x-gRNA construction

Three single guide RNAs (sgRNAs) against dSarm, each targeting a different exon, were cloned into the pU63 vector (#49410; Addgene) with the following sequences: (sgRNA1: 5′GAAGTCCATGTCGGAAATCA 3′; sgRNA2: 5′ ATCAGGCACCCTGGCCCGTT 3′; and sgRNA3: 5′ GAAGCCCTCTCACTCCGCAC 3′). This multiplexed construct was sent to BestGene Inc. (Chino Hill, CA) for targeted insertion into the attp2 site on the third chromosome. These constructs and flies were generated and shared by Dion Dickman's lab.

### CRISPR/Cas9 gene editing for dSarm

dSarm-3x-gRNA flies were crossed to either M12-GAL4 (expressed in two motoneurons per hemisegment for injuries), D42-GAL4 (pan-motoneuron expression for NMJ measurements), or BG380-Gal4 (pan-motoneuron expression for *puc*-LacZ reporter) with UAS-Cas9 to induce active somatic CRISPR mutagenesis.

### Larvae rearing

All larvae were reared on yeast-agar food at 29°C.

### Nerve crush injury

To study degeneration, we made use of a previously described nerve crush assay (Xiong et al., 2010). Early third instar larvae were anesthetized in a PBS ice bath for 20 min before being placed on an inverted petri dish. Dumostar number 5 forceps were used to pinch the larval nerves through the cuticle to damage the axons. Injured larvae were placed in a petri dish with yeast-agar food and incubated at 29°C for the indicated time lengths.

### Dissections and staining

Larva were placed in 1xPBS on ice 20 min prior to dissection. After dissections, larval filets were fixed with 4% paraformaldehyde at RT for 20 min and then washed three times with 1xPBS. Fixed filets were then blocked with 5% normal goat serum (NGS) diluted in 1xPBS with 0.25% Triton X (1xPBST) at room temperature (22C) for 1 h and then were stained with antibodies in 5% NGS in 1xPBST. Samples were incubated with primary antibodies overnight at 4°C, while secondary antibody staining was done at room temperature for 2 h (with three 10 min 1xPBST washes between antibodies). Following three 10-min washes with 1xPBST, all samples were then mounted using Prolong Diamond mounting media and were given at least 24 h to set before imaging. Primary antibodies used in this study were as follows: Rat anti-mCD8 (Invitrogen MCD0800), Mouse anti-β-Galactosidase (DSHB 40-1A), Mouse anti-GFP (Invitrogen 3E6), Mouse anti-CSP (DSHB AB49), Mouse anti-Futsch (DSHB 22C10), and Rabbit anti-HA (Cell Signaling 3724s). Secondary (including conjugated) antibodies used in this study were as follows: 488 Rabbit anti-GFP (Fisher), Cy3 anti-Rat (Fisher), 568 anti-Mouse (Fisher), 488 anti-Mouse (Fisher), and 647 anti-Rabbit (Jackson).

### Microscopy

All images were taken on a spinning disk confocal microscope (Improvision) with a C9100-50 EMCCD camera (Hamamatsu), a Nipkow CSU scanner (Yokogawa), and an Axio Observer (Zeiss). All conditions/genotypes within a repeat of an experiment were imaged at the same time with the same laser and capture settings to limit signal variation due to equipment. Volocity software (Quorum Technologies) was used for all imaging and quantification.

### Injury-induced degeneration scoring

Axon motoneuron pairs were labeled by M12 GAL4-expressed mCD8-GFP (or RFP), and scoring was done on fixed and stained larval filets. Conditions were scored blind using the following scale: completely intact (0%), continuous with varicosities (33%), partially fragmented and partially continuous (66%), and fully degenerated (100%). Sample sizes reported are the total number of axon pairs scored (M12-Gal4 labels two neurons per nerve, whose are scored together) from at least five animals per condition.

### Sarm1-TIR-induced degeneration and clearance scoring

UAS-Sarm1-TIR was co-expressed with UAS-mCD8-RFP in pairs of motoneuron axons using the m12-Gal4 driver. Axon pairs from the ten nerves innervating segments A3–A7 of the larva were scored (20 axons total) on the following scale: continuous or mostly continuous (0%), fully degenerated (50%), and cleared (100%). A single average value was calculated for each larva.

### *puc*-LacZ JNK reporter

Third instar larvae containing the *puc-*LacZ reporter (Ring and Arias, 1993) were dissected, fixed, and stained for β-galactosidase. Only male larvae were used due to the driver (BG380-GAL4) being on the X chromosome. Midline nuclei in the ventral nerve cord (VNC) corresponding to motoneurons that innervate segments 4–7 of the larvae were quantified, with 8–10 nuclei being summed within each section. Background readings were taken on either side of each midline motoneuron cluster, avoiding other stained nuclei (3 values per section, 12 total per animal). Values are relative to the mean of the control animals (normalized).

### Neuromuscular junction quantifications

Between one to three Muscle 4, NMJs per larva in abdominal segments 3–5 were imaged at 40x using confocal microscopy and analyzed using Volocity software (Quorum Technologies). Each NMJ was manually outlined, and the region of interest was copied to a nearby empty area of muscle to obtain a background reading. The total intensity of membrane maker and CSP staining within each ROI and equivalent background were summed and subtracted. All reported values are relative to the membrane marker/synaptic staining intensity of the control condition. NMJs are plotted individually from at least five total animals per condition.

### Tagged protein quantifications (nerves)

The intensity of dSarm-GFP in the axons/nerves was measured by imaging immediately posterior of the nerve cord in fixed and stained larva. Three nerves corresponding to segments 5–7 of the larvae were stamped with five 50 μm diameter ROI cylinders (for 15 total readings per animal, averaged into a single value). Nine background readings were taken from around the nerves and were averaged and subtracted from the mean nerve reading, and then the ratio of the tagged protein to the membrane marker was calculated. All reported values are relative to the level of the normalized control condition. Each data point is the average from a single larva.

### Quantification of dSarm-GFP

UAS-dSarm-GFP was coexpressed with UAS-mCD8-RFP in MNSNc motoneurons using the m12-Gal4 driver. Images of the ventral nerve cord and exiting segmental nerves were imaged at 40x magnification by confocal microscopy. Cell bodies were quantified by manually outlining ROIs around motoneuron soma pairs from sections of the VNC that innervate segments 5–7 of the larval body. Background readings were made by dragging the ROIs to empty areas around the VNC. Measurements of the axons corresponding to these neurons were done by stamping axon pairs with five 20 μm circular ROIs (15 readings total) with three background readings around each axon pair (nine total). Each data point represents the averaged measurements acquired from three motoneuron pairs in a single larva.

## Results

### dSarm is downstream of Raw in regulating axonal degeneration

**Figure 1:**
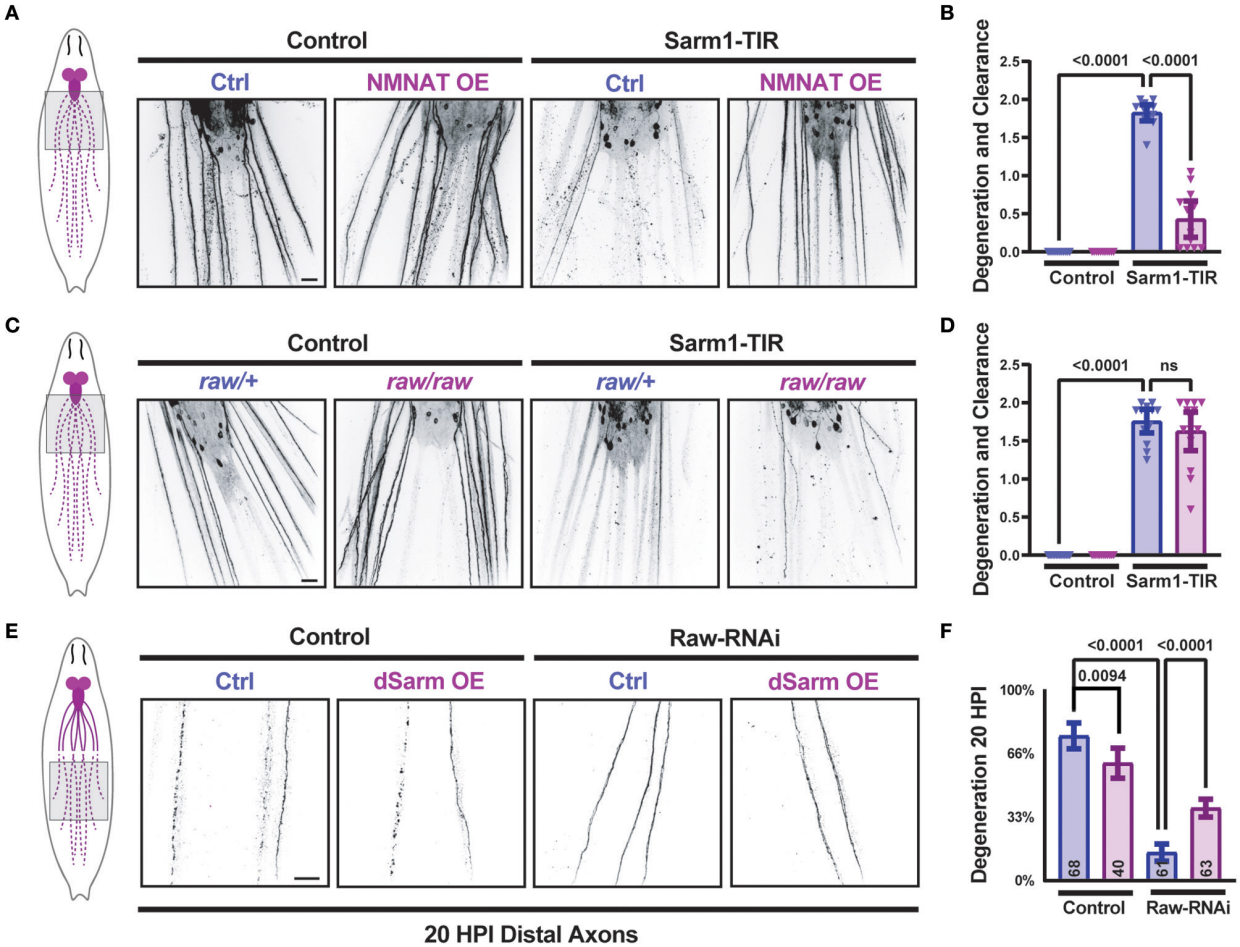
dSarm acts downstream of Raw in regulating axon degeneration. *Drosophila* motoneuron axons are visualized in the segmental nerves of third instar larvae by expression of the membrane-targeted UAS-mCD8::GFP reporter with the m12-Gal4 driver, which drives expression in the MNSNc neurons (Ritzenthaler et al., 2000). To control the number of UAS elements, animals expressed either UAS-luciferase (control) or UAS-Sarm1-TIR, and UAS-lexA RNAi (ctrl) or UAS-NMNAT. **(A, B)** Expression of the Sarm1 catalytic TIR domain leads to spontaneous degeneration of MNSNc axons in the third instar larvae; this is rescued in neurons that co-express the UAS-dNMNAT transgene. **(C, D)** In contrast, mutations in *raw, (raw*^*dcp*−1^*/raw*^134.47^), which inhibit Wallerian degeneration similarly to *raw*-RNAi (Hao et al., 2019), have no effect on the degeneration caused by the expression of Sarm1-TIR. **(E, F)** Expression of UAS-dSarm alone did not enhance the rate of Wallerian degeneration but instead caused a mild delay in degeneration. In contrast, co-expression of dSarm partially restores degeneration to *raw*-RNAi axons. **(F)** Quantification of axonal degeneration was scored blinded using visual scales described in Materials and Methods. Scale bars are 20 μm. The one-way ANOVA statistical test with Tukey correction for multiple comparisons was used for statistical comparisons. Error bars show 95% confidence interval.

We previously discovered that Raw promotes the degeneration of injured axons in *Drosophila*. When Raw is mutated or knocked down, Wallerian degeneration of both motor and sensory neuron axons is strongly inhibited (Hao et al., 2019). To investigate the relationship between Raw and dSarm, we first considered the possibility that Raw regulates axonal degeneration downstream of dSarm's catalytic activity. We expressed the self-dimerizing catalytic TIR domain from human Sarm1 (Gerdts et al., 2015) in *Drosophila* motoneuron pairs using the M12-Gal4 driver. Larval axons and NMJ nerve terminals in TIR-expressing MNs underwent spontaneous degeneration, which was visible in third-instar larvae (Figures 1A, B). Consistent with previous findings (Gerdts et al., 2015), the degeneration induced by the catalytic Sarm1 TIR domain could be inhibited by co-expression of a UAS-dNMNAT transgene [encoding the *Drosophila* nicotinamide mononucleotide adenylyltransferase enzyme (Zhai et al., 2006)] (Figures 1A, B). While mutations in *raw* (*raw*^*dcp*−1^*/raw*^134.47^) cause a significant delay in the degeneration of injured axons (Hao et al., 2019), *raw* mutations had no impact on the degeneration of TIR-expressing MN axons (Figures 1C, D). These observations suggest that Raw is unlikely to influence degeneration at a step downstream of the catalytic activity of dSarm. Instead, these data favor a role for Raw either upstream or in parallel to the action of dSarm in promoting axonal degeneration.

In contrast to the TIR domain alone, ectopic overexpression full-length dSarm-GFP (using the same expression system) does not lead to spontaneous degeneration (Figures 1E, F). However, this method of elevating dSarm levels led to partial rescue of the delayed degeneration caused by loss of Raw (Figures 1E, F). This suggests that at least part of Raw's actions in promoting Wallerian degeneration may occur upstream of dSarm, potentially by influencing its levels, localization, and/or catalytic activity.

### Raw promotes axon localization of dSarm

**Figure 2:**
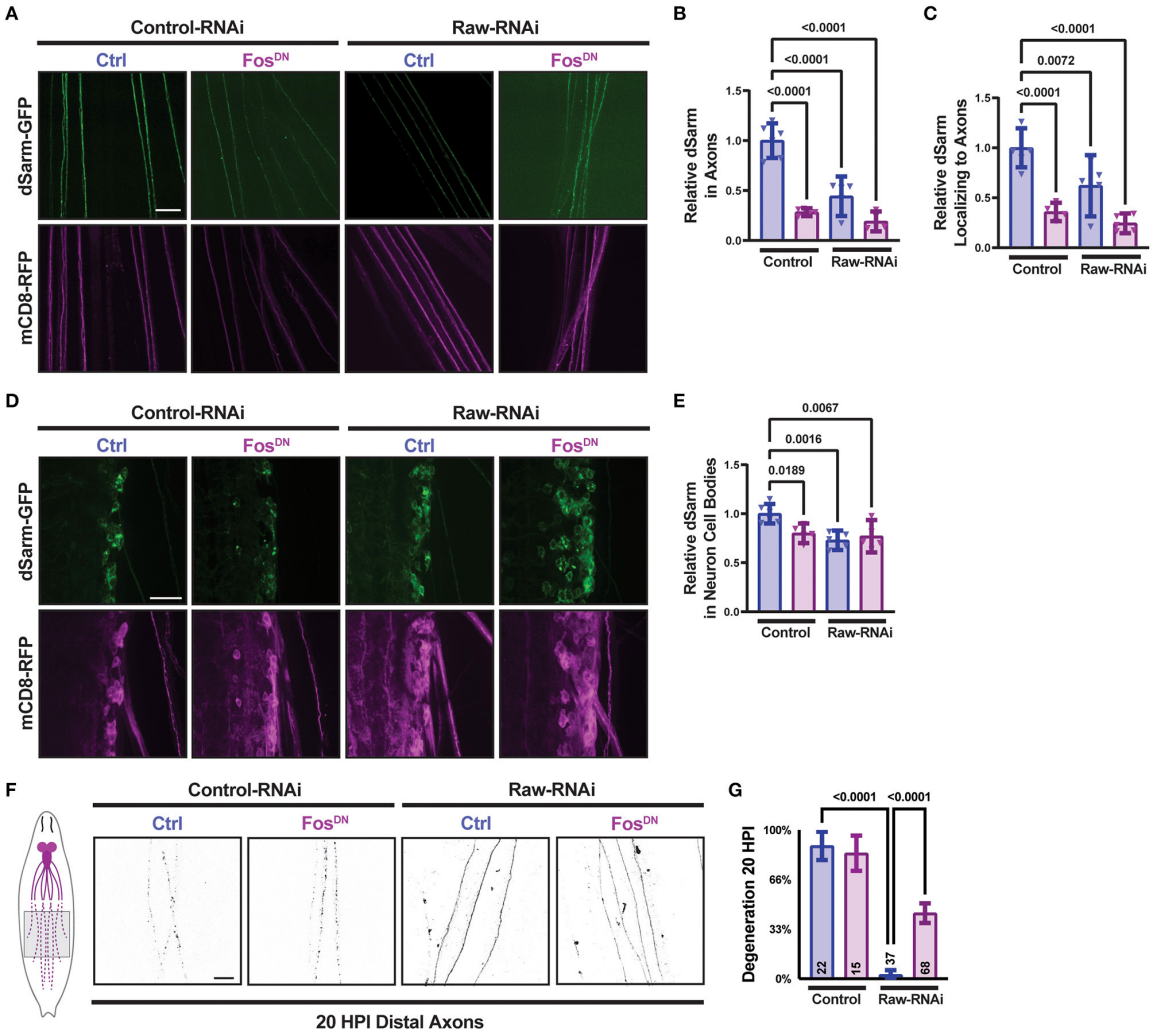
Raw promotes axon localization of dSarm. UAS-dSarm-GFP was coexpressed with the membrane-targeted UAS-mCD8-RFP reporter to label neuronal membrane. In this background, UAS-Raw RNAi was compared to UAS-lexA RNAi (control-RNAi), together with either UAS-Fos-DN or UAS-luciferase (ctl), using the M12-Gal4 driver. UAS-Dcr2 was included to enhance RNAi efficiency; hence, all compared animals contained five UAS transgenes. **(A)** Representative images of segmental nerves showing the axonal localization of dSarm-GFP. **(B)** Mean intensity of dSarm-GFP measured in axons. **(C)** Intensity of axonal dSarm-GFP relative to dSarm-GFP measured in cell bodies [from **(D, E)**]. **(D)** Example images of MNSNc cell bodies within the ventral nerve cord. **(E)** Quantification of mean dSarm-GFP within cell bodies. **(F)** Representative images of MNSNc axons undergoing Wallerian degeneration at 20 h postinjury (HPI). Axons that express *raw* RNAi show a delay in Wallerian degeneration compared to control RNAi. Co-expression of Fos-DN [which contains the DNA binding domain of Fos but lacks the transcription activating region and acts as a dominant-negative inhibitor of Fos (Eresh et al., 1997)], partially restores the degeneration of *raw*-RNAi axons. **(G)** Axonal degeneration was scored blinded using visual scales described in Materials and Methods. Scale bars are 20 μm. A one-way ANOVA statistical test with Tukey correction for multiple comparisons was used for statistical comparisons. Error bars show 95% confidence interval.

Therefore, we next asked whether manipulations to Raw had any effect on the levels or localization of a transgenically expressed dSarm-GFP. We imaged dSarm-GFP coexpressed with mCD8-RFP in MNSNc axons and cell bodies in parallel for different genetic conditions by confocal microscopy (Figures 2A–E). Compared to MNs in animals expressing UAS-control (lexA) RNAi, we observed that MNs depleted of Raw had reduced intensity of dSarm-GFP in their axons (Figures 2A, B). The levels of dSarm-GFP in cell bodies were similar in all genotypes (Figures 2D, E); hence, the ratio of axonal/cell body localized dSarm was reduced in raw-RNAi depleted MNs (Figure 2C).

The regulation of dSarm localization is an attractive potential mechanism for Raw's role in axonal degeneration. However, a simple model that Raw promotes degeneration by promoting dSarm localization is not consistent with other genetic data. We previously found that the delay in degeneration in *raw*-deficient neurons was rescued by the expression of a dominant negative inhibitor of Fos, Fos^DN^ [(Hao et al., 2019), Figures 2F, G]. Counter-intuitively, expression of Fos^DN^ led to a decrease in axonal dSarm-GFP levels (Figures 2A, C). Therefore, the localization of dSarm-GFP in axons does not consistently predict the rate of axonal degeneration. Taken together, these data imply the existence of multiple points of regulation for dSarm: both Raw and Fos regulate the levels of dSarm in axons, but signaling downstream of Fos influences axonal degeneration independently of dSarm localization. This is consistent with both dSarm overexpression (Figures 1E, F) and Fos inhibition (Figures 2F, G), each only partially restoring degeneration in *raw* deficient axons.

### Raw and dSarm regulate synaptic structure

**Figure 3:**
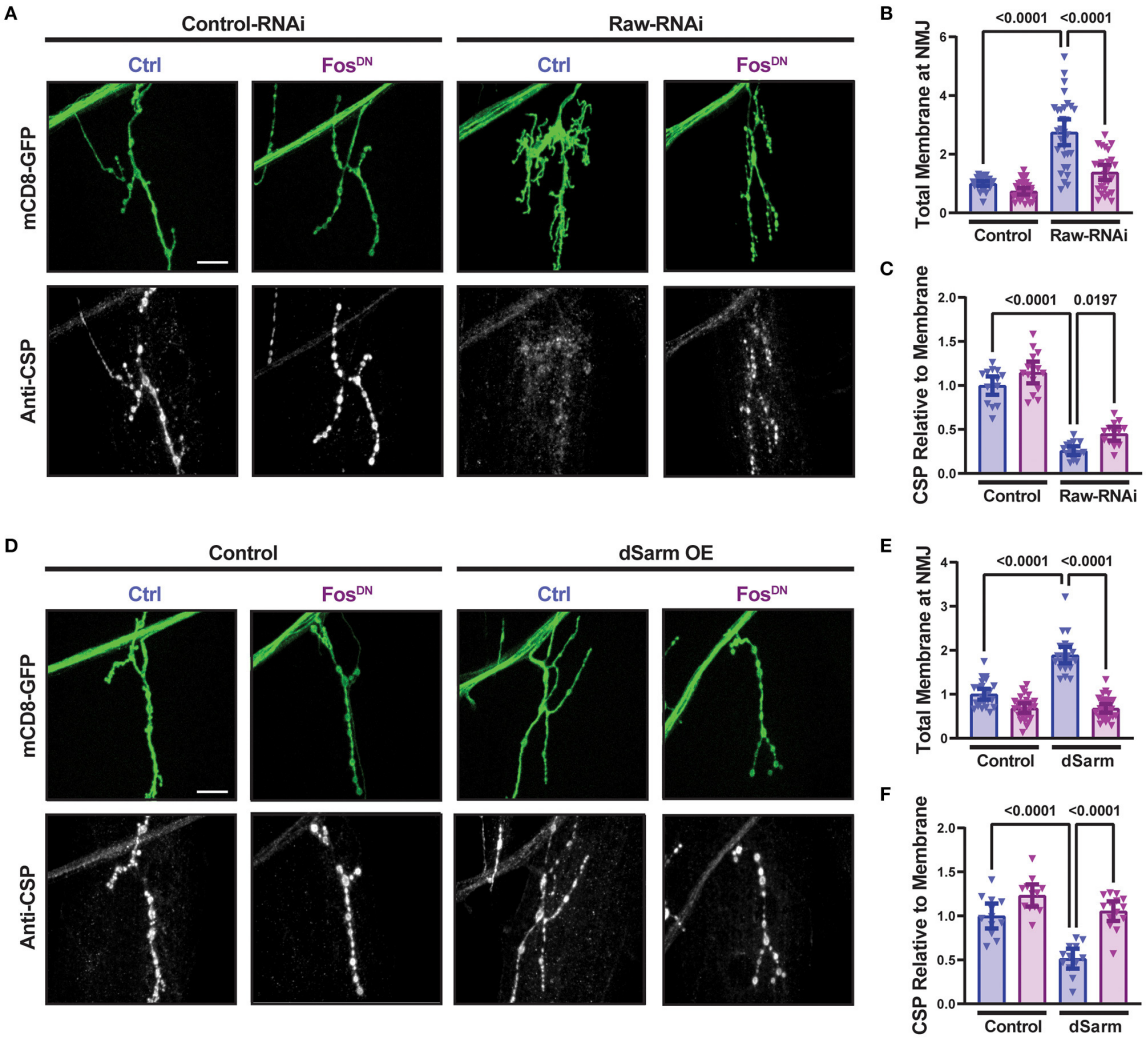
Raw and dSarm regulate NMJ structure and synaptic protein levels. **(A–C)** Knockdown of *raw* results in overgrowth and altered structure at the Muscle 4 neuromuscular junction (NMJ) as well as a reduction in the relative levels of cysteine-string protein (CSP); the overgrowth (measured as total membrane marker intensity at the NMJ) shows a strong dependence on Fos. **(D–F)** Overexpression of dSarm results in overgrowth at the NMJ [matching similar previous findings (McLaughlin et al., 2016; Brace et al., 2022)] and relative reduction in CSP. Both show a strong dependence on Fos. The driver for all panels is D42-Gal4, with Dcr2 expressed for RNAi efficiency. All scale bars are 20 μm. One-way ANOVA statistical test with Tukey correction for multiple comparisons was used for panels **(B, C, E, F)**. Error bars show 95% confidence interval.

In addition to promoting axonal degeneration, dSarm is known to participate in intracellular signaling pathways [reviewed in (Waller and Collins, 2022)]. In *Drosophila* motoneurons, dSarm regulates a signaling pathway that controls the structure of presynaptic terminals (McLaughlin et al., 2016; Brace et al., 2022). We therefore asked whether Raw modulates the presynaptic defects caused by misregulated dSarm. RNAi-knockdown of *raw* in motoneurons (using D42-Gal4) results in altered NMJ morphology that is Fos-dependent (Figure 3A). NMJ terminals formed by *raw*-depleted neurons showed extensive filopodia-like branches that lacked Futch (Supplementary Figure 1), a marker of stable microtubules (Chang and Balice-Gordon, 2000). Since boutons could not be clearly defined and counted, we measured the total intensity of membrane-targeted UAS-mCD8-GFP (Figures 3A, B); this was greatly increased at *raw*-RNAi NMJs but suppressed in *raw*-RNAi; Fos^DN^ co-expressing neurons. Concomitant with filopodial structures, *raw*-RNAi NMJs showed a Fos-dependent reduction in levels of synaptic vesicle proteins, shown with cysteine-string-protein (CSP) in Figures 3A, C. These observations imply that Raw controls a Fos-dependent signaling pathway that regulates the growth and structure of the presynaptic nerve terminal.

The dramatic NMJ phenotypes caused by RNAi knockdown of Raw are strikingly different from our prior observations with *raw* hypomorph mutants, for which we observed no defect in synaptic structure or physiology (Hao et al., 2019). We therefore tested whether they are indeed due to Raw function by asking whether they could be rescued by co-expression of Raw-GFP cDNA. Co-expression of Raw-GFP but not of a control UAS line successfully rescued the synaptic overgrowth and increased CSP expression (Supplementary Figure 2). We infer that Raw functions to control the structure of the NMJ terminal.

**Figure 4:**
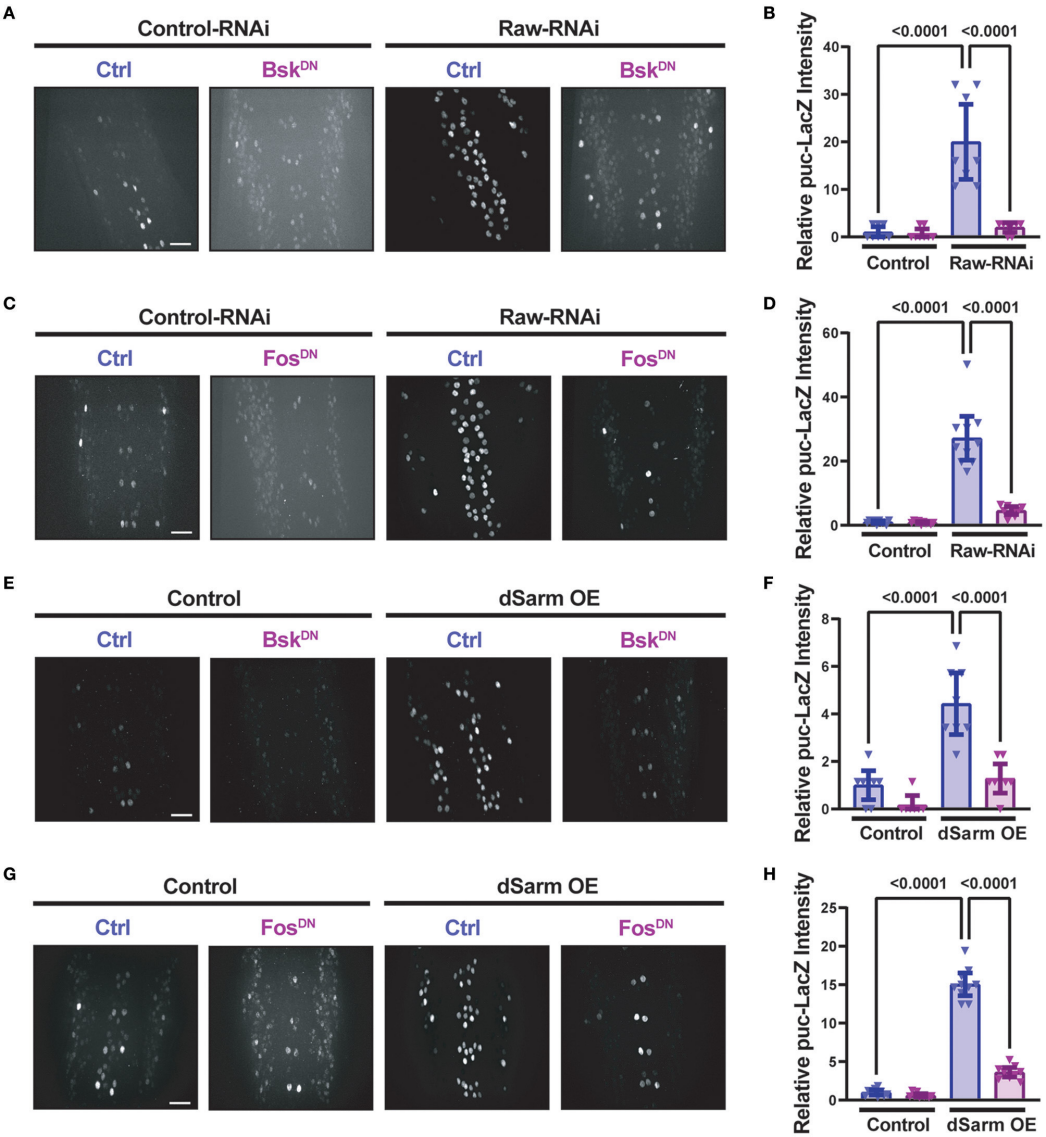
Raw and dSarm have opposing influences on a kinase signaling pathway. Beta-galactosidase expression in motoneuron cell bodies in the dorsal midline of the ventral nerve cord of larvae containing the *puc*-lacZ reporter for JNK signaling (Martín-Blanco et al., 1998). In all conditions, BG380-Gal4 was used to drive the expression of a similar number of control and experimental UAS lines together with UAS-Dcr2 to aid RNAi efficiency. All compared animals are male hemizygous for BG380-Gal4 on X. **(A, B)** To evaluate the effect of inhibiting JNK on *puc*-lacZ expression, UAS-Raw-RNAi or UAS-LexA-RNAi (control-RNAi) was coexpressed with either UAS-BsK^DN^ or UAS-Luciferase (ctrl). **(C, D)** To evaluate the effect of Fos inhibition, UAS-Raw-RNAi or UAS-LexA-RNAi (control-RNAi), was coexpressed with either UAS-Fos^DN^ or UAS-Luciferase (ctrl). **(E, F)** The requirement for JNK in *puc*-lacZ induction was evaluated in UAS-dSarm-GFP vs. UAS-*lexA*-RNAi (control), coexpressed with either UAS-BsK^DN^ or UAS *luciferase-RNAi* (ctrl). **(G, H)** The requirement for Fos was evaluated for UAS-dSarm-GFP vs. UAS-Luciferase (control), coexpressed with either UAS-Fos^DN^ or UAS-*lexA-RNAi* (ctrl). Scale bars are 20 μm. The one-way ANOVA statistical test with Tukey correction for multiple comparisons was used for statistical comparisons. Error bars show 95% confidence interval.

Similarly to *raw* knockdown, overexpression of dSarm also led to a Fos-dependent synaptic overgrowth phenotype (McLaughlin et al., 2016; Brace et al., 2022). We observed that this dSarm overexpression phenotype includes increased mCD8-GFP levels and reduced levels of CSP (Figures 3D–F). In addition, both manipulations lead to elevations in the expression of the *puc*-lacZ reporter for JNK signaling (Hao et al., 2019; Brace et al., 2022, and Figure 4). For both *raw*-RNAi (Figures 4A–D) and dSarm overexpression (Figures 4E–H), *puc-*lacZ elevation is inhibited by co-expression of BskDN, a dominant-negative inhibitor of JNK signaling (Weber et al., 2000) (Figures 4A, B, E, F), and by FosDN, a dominant-negative inhibitor of Fos-dependent transcription (Eresh et al., 1997) (Figures 4C, D, G, H). Therefore, both Raw and dSarm regulate the structure of the presynaptic terminal, JNK MAP kinase signaling, and Fos-regulated changes in gene expression.

### dSarm is not downstream of Raw in the regulation of synaptic growth

**Figure 5:**
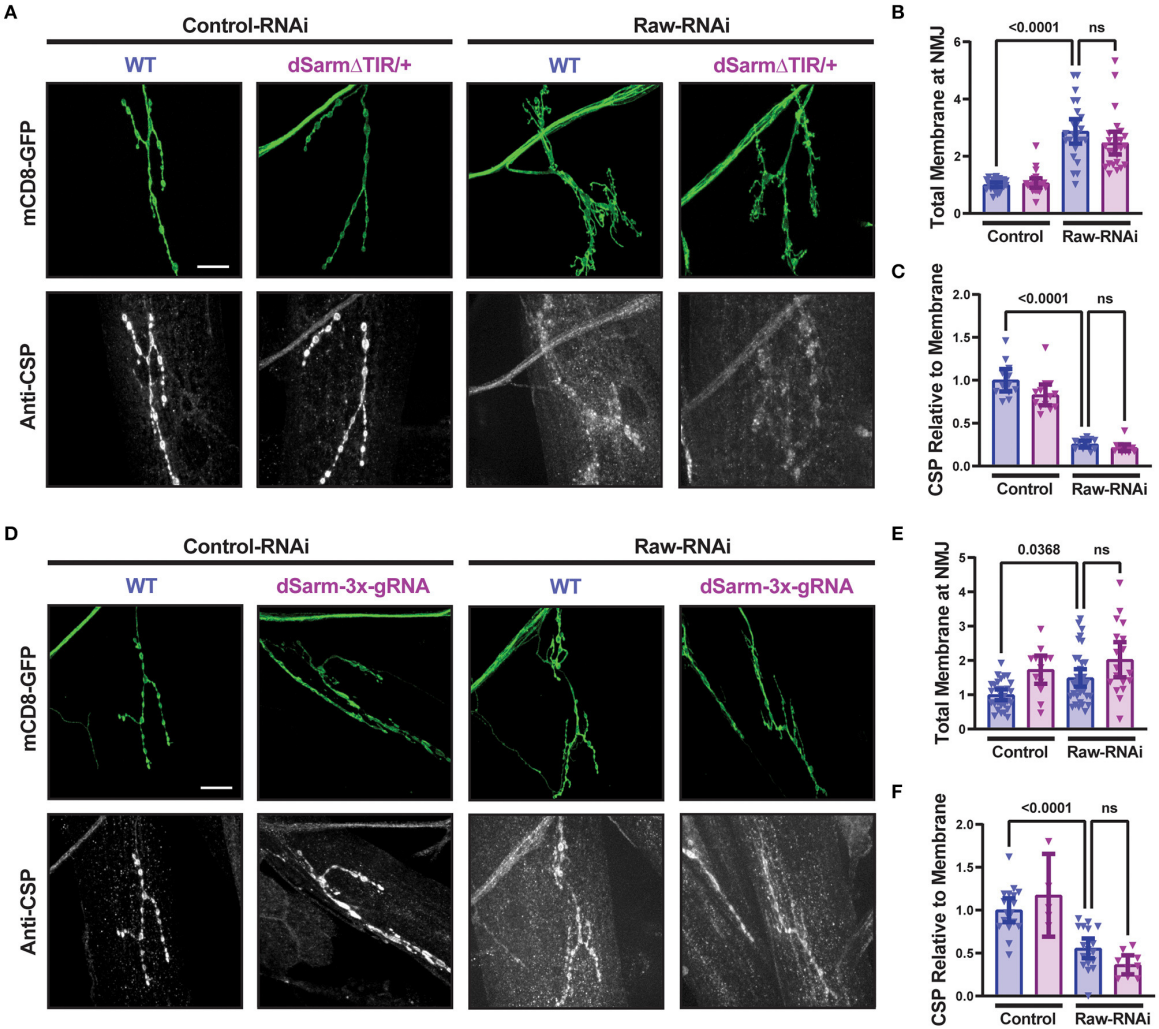
dSarm is not downstream of Raw in regulating the NMJ. Presynaptic membranes of neuromuscular junctions are visualized by expression of UAS-mCD8-GFP using D42-Gal4, with co-staining of cysteine string protein (CSP) for visualization and quantification of synaptic vesicles. Dcr2 co-expressed for RNAi efficiency. **(A–C)** UAS-*raw*-RNAi is used to knockdown *raw* in comparison to UAS-*lexA*-RNAi (control-RNAi) in either a wildtype (WT) or dSarm LOF mutant heterozygote background [which is sufficient for delaying degeneration, (Herrmann et al., 2022)] and Supplementary Figure 2A]. **(D–F)** UAS-*raw-RNAi* or UAS–*lexA*-RNAi (control) was expressed *via* D42-Gal4 in either WT motoneurons or in motoneurons with cell-specific Cas9-mediated knockout of the dSarm locus using the dSarm-3x-guide RNA. Cas9 is expressed in panels D-F for gRNA-mediated gene knockout. All scale bars are 20 μm. The one-way ANOVA statistical test with Tukey correction for multiple comparisons was used for panels **(B, C, E, F)**. Error bars show 95% confidence interval.

**Figure 6:**
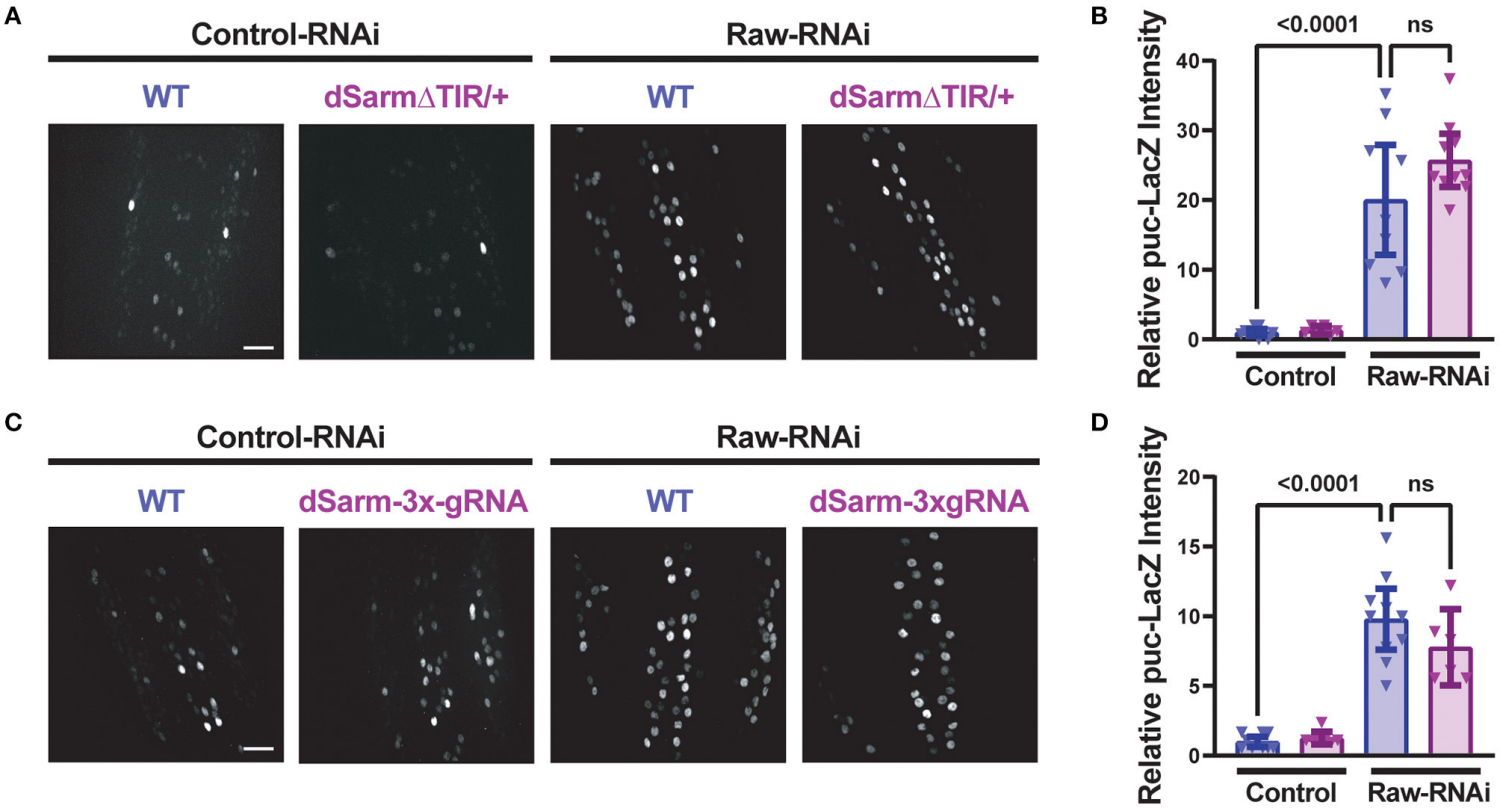
Raw-regulated nuclear JNK signaling persists following disruption of dSarm. Beta-galactosidase expression in motoneuron cell bodies in the dorsal midline of the ventral nerve cord for beta-galactosidase in larvae containing the *puc*-lacZ reporter for JNK signaling. The BG380-Gal4 driver was used in hemizygous males to drive an equal number of UAS-transgenes in all conditions, together with UAS-Dcr2 for RNAi efficiency. Cas9 is expressed in panels **(C, D)** for gRNA knockout. **(A, B)** dSarm function was inhibited in dSarmΔTIR heterozygous mutants, which inhibit Wallerian degeneration (Herrmann et al., 2022) *raw*-RNAi vs. *lexA*-RNAi (control-RNAi) animals. **(C, D)** CRISPR/Cas9 knockdown of dSarm, which inhibits Wallerian degeneration (Supplementary Figure 3B), was evaluated for its effect on *puc*-lacZ expression in Raw-RNAi vs. *lexA*-RNAi (control-RNAi) animals. Scale bars are 20 μm. The one-way ANOVA statistical test with Tukey correction for multiple comparisons was used for statistical tests. Error bars show 95% confidence interval.

To better understand the opposing relationship of Raw and dSarm in synapse regulation, we asked whether dSarm is required for the synaptic overgrowth phenotype of *raw* knockdown in motoneurons. We used two different genetic reagents to inhibit dSarm function. First, we used a dominant-negative allele of dSarm (Herrmann et al., 2022), referred to here as dSarm-DeltaTIR. This Crispr/Cas9 engineered mutation replaces endogenous dSarm with dSarm-Delta-TIR, which retains dSarm's ARM and SAM domains but lacks the catalytic TIR domain. dSarm-DeltaTIR homozygous animals die as third-instar larvae (Herrmann et al., 2022). However, since the ARM and SAM domains facilitate oligomerization and regulation of the holoenzyme (Gerdts et al., 2013; Sporny et al., 2019, 2020; Bratkowski et al., 2020; Jiang et al., 2020; Shen et al., 2021), dSarm-DeltaTIR can dominantly inhibit endogenous dSarm function; dSarm-DeltaTIR/+ heterozygotes show a delay in Wallerian degeneration of injured olfactory neuron axons (Herrmann et al., 2022) and motoneuron axons (Supplementary Figure 3A). The second reagent was a dSarm-targeted guide RNA; co-expression of dSarm-gRNA with UAS-Cas9 (driven by M12-Gal4) led to the expected phenotype for somatic knockout of dSarm of delayed Wallerian degeneration (Supplementary Figure 3B). We then introduced dSarm-DeltaTIR and dSarm gRNA into the background of *raw*-RNAi expressing motoneurons (Figure 5). Neither manipulation to inhibit dSarm altered the NMJ defects caused by *raw* knockdown (Figure 5). Similarly, the elevated *puc*-lacZ expression in *raw-*RNAi neurons was also not affected by knockout or heterozygous mutations in dSarm (Figure 6). Therefore, the regulation of synaptic structure by Raw does not require dSarm and hence may occur downstream or independently of dSarm.

### Raw regulates synaptic growth independently of the Ask1 kinase

**Figure 7:**
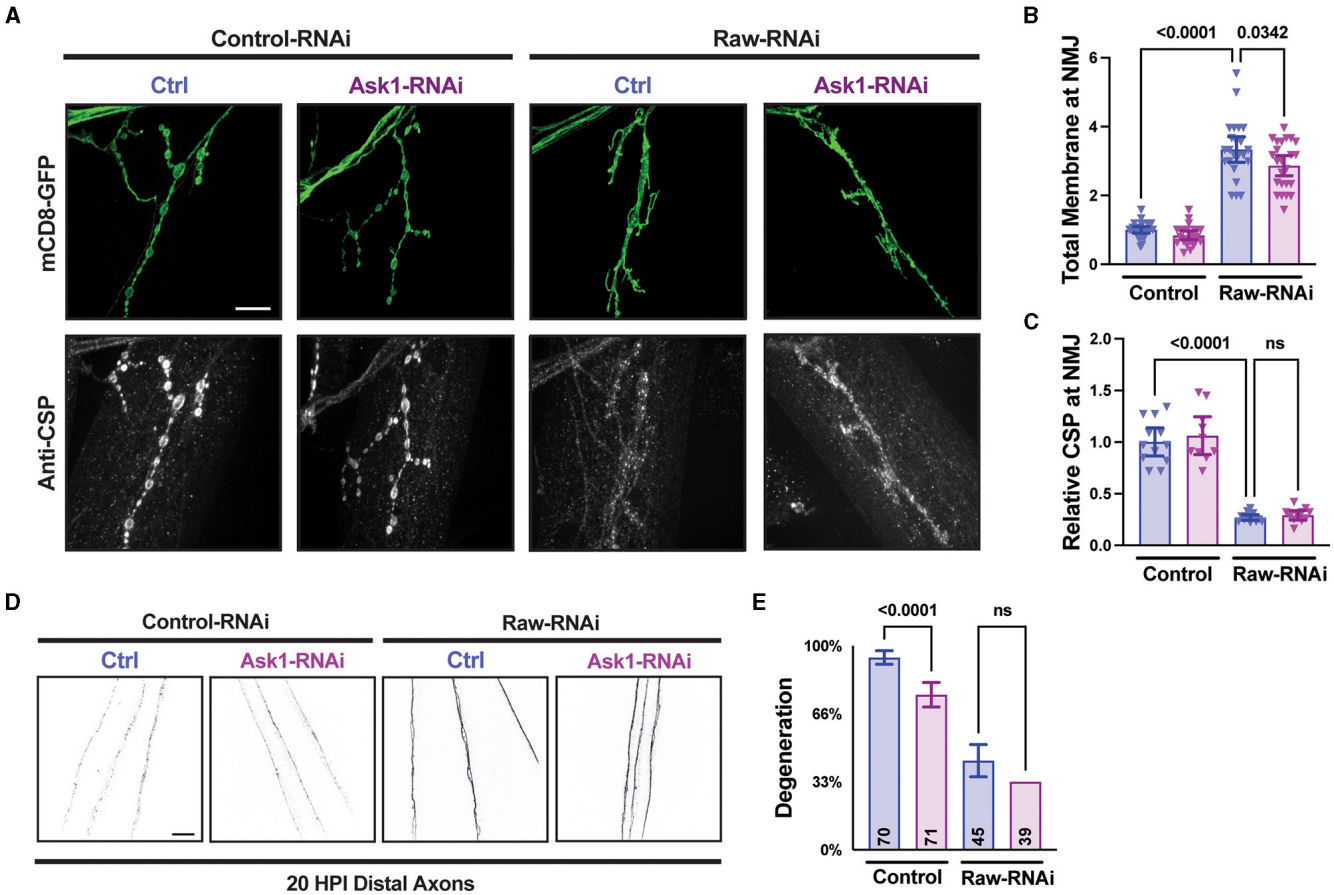
Raw acts independently of Ask1 in regulating degeneration and NMJ structure. **(A–C)** NMJ measurements taken from Muscle 4 within segments 3–5 of the larval body wall. UAS-*raw*-RNAi or UAS-*lexA*-RNAi (control-RNAi) was expressed with either UAS-*ask1*-RNAi or UAS-*luciferase*-RNAi (ctrl) along with UAS-Dcr2 and UAS-mCD8-GFP using D42-Gal4 as the driver. Larvae were co-stained for cysteine string protein (CSP) to label synaptic vesicles. The *ask1* RNAi line was used previously to show that Ask1 acts downstream of dSarm in regulating NMJ growth (Brace et al., 2022). **(D, E)** Distal severed axons 20 h post-injury (HPI) in third instar larvae expressing UAS-*raw*-RNAi or UAS-*lexA*-RNAi (control-RNAi) with either UAS-*ask1*-RNAi or UAS-*luciferase*-RNAi (ctrl) along with UAS-Dcr2 and UAS-mCD8-GFP using M12-Gal4 as the driver. Scale bars are 20 μm. The one-way ANOVA statistical test with Tukey correction for multiple comparisons was used for panels **(B, C, E)**. Error bars show 95% confidence interval.

**Figure 8:**
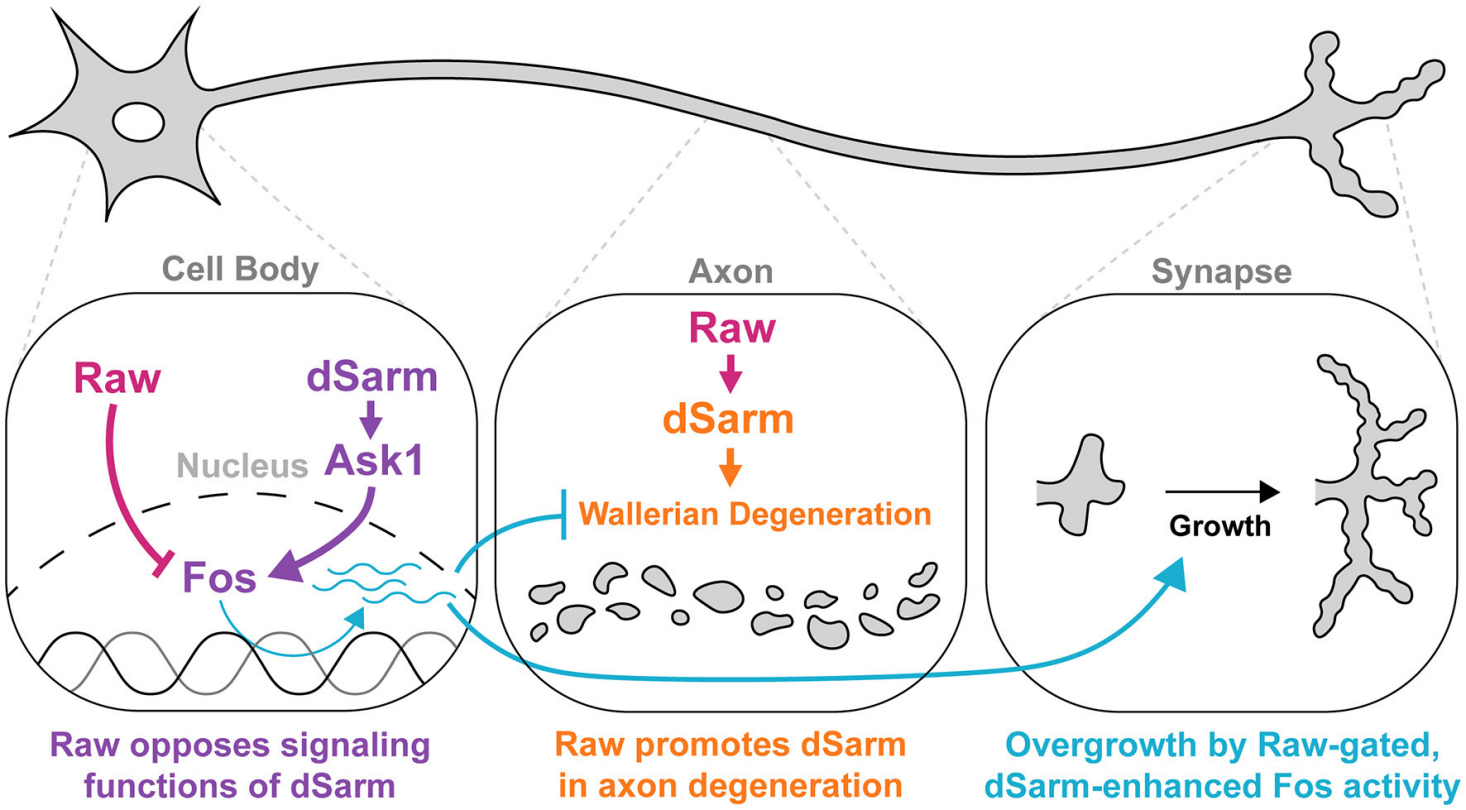
Raw and dSarm have distinct functional relationships in the regulation of synaptic growth and axonal degeneration. First, Raw opposes a signaling function of dSarm (in purple), which promotes Fos-dependent expression of genes that influence the resiliency of axons to degeneration and synaptic growth (turquoise). This pathway is restrained under normal conditions (in wild-type uninjured neurons); however, the loss of Raw or over-expression of dSarm leads to its activation. The Fos-dependent genes are thus far not known but are inferred to be co-regulated with the *puc*-lacZ reporter. In the middle panel, Raw has a contrasting relationship with dSarm in promoting (not opposing) axonal degeneration, shown in orange. In this case, Raw promotes the localization of dSarm to axons where its catalytic activity leads to metabolic rundown and Wallerian degeneration.

Previous studies of dSarm and its *C. elegans* homolog TIR-1 have identified the MAPKKK Ask1 as an important mediator of downstream signaling (Chuang and Bargmann, 2005; Brace et al., 2022; Ding et al., 2022). To further understand the relationship between Raw and dSarm, we asked whether the axonal and synaptic phenotypes of Raw were also dependent upon *Ask1*. To inhibit *Ask1*, we used a UAS-*Ask1* RNAi line that has been previously shown to strongly suppress synaptic overgrowth in dSarm over-expressing animals (Brace et al., 2022). This RNAi line showed a mild inhibition to axonal degeneration on its own but failed to alter the axonal degeneration or synaptic phenotypes caused by loss of Raw (Figure 7). These observations further suggest that Raw regulates synaptic growth independently (and potentially downstream) of dSarm/Ask1 signaling (Figure 8).

## Discussion

In a previous study, we discovered that the transmembrane protein Raw regulates axonal degeneration by regulating a nuclear signaling pathway (Hao et al., 2019). In this study, we considered the potential mechanistic relationship between Raw and dSarm, the *Drosophila* ortholog of the SARM1 (sterile alpha and TIR motif-containing protein 1) enzyme. In addition to its central role in driving metabolic catastrophe and degeneration in axons by breaking down NAD+, SARM1 functions as an evolutionarily conserved scaffold of MAP kinase signaling (Waller and Collins, 2022). In *C. elegans*, the Raw ortholog OLRN-1, functionally antagonizes the UNC-43–TIR-1–ASK1–NSY-1 MAP kinase signaling pathway, engaged by the SARM1 ortholog TIR-1 (Chuang and Bargmann, 2005; Bauer Huang et al., 2007; Inoue et al., 2013; Foster et al., 2020).

### Raw antagonizes a dSarm-mediated signaling pathway that regulates synaptic growth

Here, we found that Raw antagonizes a functional output of dSarm-triggered signaling at the *Drosophila* larval NMJ. Specifically, both RNAi knockdown of Raw and overexpression of dSarm lead to synaptic overgrowth phenotypes that require the JNK MAP kinase and Fos transcription factor and coincide with the induction of a transcriptional reporter for JNK signaling (*puc*-lacZ). These observations imply the existence of a transcriptional state that is induced by dSARM-mediated signaling and restrained by the action of Raw.

This state is associated with the growth of the synaptic terminal at the expense of synaptic organization. In Raw-RNAi-depleted neurons, synaptic boutons and synaptic vesicle-associated proteins are barely detectable, while the labeled axonal membrane is strongly increased. The total membrane surface area of the NMJ terminus is increased, suggesting a state of membrane growth. dSarm over-expression induces similar, though milder, synaptic phenotypes; this may be due to strong restraint by Raw under normal conditions. While this state is normally restrained in larval motoneurons, we speculate that it may be engaged in a developmental context since the growth of the axon and maturation of synaptic structures are tightly orchestrated during circuit development. In support of this idea, a recent study found that restraint of dSarm/SARM1 function by Wnk kinases (in both *Drosophila* and mammalian neurons) influences the branching and stability of axons (Izadifar et al., 2021), suggesting an instructive role for SARM1 and its regulation during development. Whether these functions are also mediated by the ASK1 kinase, Fos transcription factor, and/or inhibited by Raw is a topic for future study.

Synaptic growth and axonal degeneration may be mechanistically coupled at the level of cell adhesion. It is interesting that developmental pruning of *Drosophila* axons requires destabilization of adhesion regulated by JNK signaling (Bornstein et al., 2015), while a previous study has shown that Raw regulates adhesion (together with JNK signaling) during gonad morphogenesis (Jemc et al., 2012). A functional target of Raw and JNK during gonad development, DE-cadherin, did not modify the axonal degeneration or synaptic overgrowth phenotypes for *raw* (TJ Waller and CA Collins, unpublished observations); however, JNK may regulate different adhesion proteins in axons [including Fas II, (Bornstein et al., 2015)].

How might Raw mechanistically antagonize dSarm-mediated signaling? Inhibition or knockdown of dSarm did not restrict synaptic overgrowth or transcriptional reporter activation induced by loss of Raw. Similarly, RNAi knockdown of Ask1, which inhibits synaptic overgrowth caused by dSarm overexpression (Brace et al., 2022), fails to inhibit synaptic phenotypes caused by *raw* knockdown. These genetic data imply that Raw is not an upstream regulator of dSarm/Ask1-mediated signaling. Following the genetic interactions between OLRN-1 and TIR-1 in *C. elegans* (Bauer Huang et al., 2007; Foster et al., 2020), we propose that Raw restrains a separate event that is required for the execution of dSarm/Ask1 mediated signaling (Figure 8). This event would involve Fos-dependent transcription, consistent with the well-known role of Raw in antagonizing the transcriptional activity of AP-1, a downstream regulator target of JNK signaling (Bates et al., 2008; Jemc et al., 2012; Zhou et al., 2017; Luong et al., 2018; Hao et al., 2019).

The mechanism(s) by which Raw, a transmembrane protein (Lee et al., 2015) achieves these cellular functions remains a challenging question. Some structure/function studies have been undertaken for Raw and its *C. elegans* homolog OLRN-1 (Bauer Huang et al., 2007; Lee et al., 2015; Rui et al., 2020). Interestingly, the Raw-homology domain of OLRN-1 is required for its role in regulating left-right asymmetric cell fate (Bauer Huang et al., 2007), and this domain is structurally predicted to contain a TIR domain. While TIR domains mediate interactions with other TIR domains (Nimma et al., 2017), we do not see colocalization of Raw and dSarm (data not shown). Moreover, the genetic interactions observed in this study and others can be achieved by a function for Raw downstream of dSarm in the absence of any direct physical interaction. While expression of a UAS-Raw transgene can rescue *raw loss-of-function* phenotypes for axonal degeneration (Hao et al., 2019) and synaptic overgrowth (this study), we also failed to detect any apparent gain-of-function phenotypes for Raw in either the regulation of synaptic growth or axonal degeneration (TJ Waller and Yan Hao, unpublished communication). This adds further challenge to understanding the role of individual domains of Raw in these functions.

### Relationship of Raw and dSarm in the regulation of axonal degeneration

While Raw antagonizes dSarm-mediated signaling to restrain synaptic growth, it shows a puzzlingly opposite relationship with dSarm in the regulation of axonal degeneration. One aspect of this complexity may stem from the dual functions of the dSarm enzyme in regulating both acute NAD+ rundown in axons and nuclear signaling *via* Ask1/MAP kinase signaling. Nuclear signaling downstream of dSarm may not be sufficient to influence Wallerian degeneration of acutely injured axons, which are by default separated from the nucleus. However, recent studies in *C. elegans* have shown a **protective** role for TIR-1/ASK1 mediated signaling in axons (Ding et al., 2022; Czech et al., 2023). We propose that transcriptional targets of this signaling pathway, restrained by Raw/olrn-1 and activated by TIR-1/ASK1 signaling, may be functionally relevant for organizing adaptive responses to stressors in axons such as energy depletion or impairments in axonal transport. If this is the case in mammalian axons, it would be an important consideration for therapeutic strategies that target the SARM1 enzyme, particularly since the therapeutic potential of SARM1 inhibitors is aimed at inhibiting axonal loss in the context of chronic stresses rather than acutely injured axons. Identification of the downstream targets of dSARM signaling and Raw regulation that mediate axonal protection and synaptic overgrowth is an important future endeavor.

The role of Fos in the protection of injured axons complicates our understanding of additional genetic relationships between Raw and dSarm. Notably, we observed that Raw depletion leads to a reduction in the levels of dSarm-GFP localized to axons. While this could in theory provide an attractive explanation for Raw's role in promoting degeneration, we also observed that inhibiting Fos, which partially inhibits Raw's protective phenotype also leads to reduced levels of dSarm-GFP in axons. We infer that the protective state regulated by Fos in Raw-depleted neurons works independently of dSarm localization in axons. Moreover, the sheer levels of dSARM in axons *per se* are not predictive of its degenerative activity. This is consistent with recent findings that SARM1 accumulates within injured proximal axons (which do not undergo Wallerian degeneration) following spinal cord injury (Choi et al., 2022).

We note that while the synaptic overgrowth defects of *raw RNAi*-depleted neurons are fully rescued by inhibition of Fos, the protection from degeneration is only partially rescued. This leaves room for the regulation of dSarm localization, or some other yet unknown cellular action of Raw, to function additively with the Fos-dependent signaling that is restrained by Raw. In addition to counteracting MAPK signaling and restraint of AP-1, JNK and Fos-independent functions have been identified for Raw in the regulation of dendrite morphogenesis and pruning (Lee et al., 2015; Rui et al., 2020), suggesting candidate Fos-independent mechanisms for dSarm localization and/or axonal degeneration by Raw.

In summary, we found that Raw regulates at least two functional outputs of dSarm: the degeneration of injured axons and the regulation of synaptic growth. This regulation appears to involve at least two independent mechanisms. One mechanism is by antagonizing a Fos-dependent transcriptional response that is induced by dSarm and ASK1-mediated signaling (Figure 8). This relationship is saliently illustrated in the synaptic overgrowth phenotypes caused by the loss of Raw or overexpression of dSarm. In contrast, axonal degeneration may be regulated by the combined actions of dSarm's enzymatic NADase activity and its signaling functions in addition to the regulation of dSarm localization. Together, these observations suggest that Raw, dSarm, and Fos influence each other's functions through multiple points of regulation. This multiplicity may enable flexible mechanisms for neurons to orchestrate development and stress response to control the structure and resilience of axons.

## Data availability statement

The original contributions presented in the study are included in the article/Supplementary material, further inquiries can be directed to the corresponding author.

## Ethics statement

The manuscript presents research on animals that do not require ethical approval for their study.

## Author contributions

TW: Conceptualization, Data curation, Formal analysis, Investigation, Methodology, Validation, Visualization, Writing – original draft, Writing – review & editing. CC: Conceptualization, Funding acquisition, Project administration, Resources, Supervision, Writing – original draft, Writing – review & editing.

## Supplementary material

Supplementary Figure 1 Effect of *raw* knockdown on NMJ structure and Futsch. Representative images of Muscle 4 NMJs from third instar larvae. UAS-mCD8-GFP was expressed in all motoneurons by D42-GAL4. Larvae were co-stained for Futsch (22C10, DHSB). Arrows indicate filopodia-like structures. Dcr2 was expressed in all panels for RNAi efficiency. The scale bar is 20 μm. Error bars show 95% confidence interval.

Supplementary Figure 2 Overexpression of Raw rescues NMJ phenotypes of Raw RNAi. Co-expression of UAS-GFP-Raw rescues overgrowth and relative CSP decrease of Raw RNAi. **(A)** UAS-Raw-RNAi is co-expressed with UAS-mCD8-RFP and UAS-Dcr2 (for RNAi efficiency), together with UAS-GFP-Raw or UAS-Luciferase (control) *via* the D42-Gal4 driver. The scale bar is 20 μm. The two-tailed unpaired *t*-test was used for panels **(B, C)**. Error bars show 95% confidence interval.

Supplementary Figure 3 Validation of loss-of-function manipulations in dSarm. MNSNc axons are visualized using the mCD8 membrane marker transgene (tagged with either RFP or GFP) driven by m12-Gal4 and assayed for Wallerian degeneration at 20 h postinjury. **(A, B)** Heterozygous dominant negative mutations of dSarm's catalytic TIR domain, dSarmDeltaTIR/+, were compared to the control (w118) background. **(C, D)** CRISPR/Cas9 knockdown of dSarm was carried out by co-expression of dSarm-3x-gRNA with UAS-Cas9 using the m12-Gal4 driver. Control flies express a gRNA that targets the QUAS sequence (not present in these flies). The scale bars are 20 μm. The two-tailed unpaired *t*-test was used for panels **(B, D)**. Error bars show 95% confidence interval.

## References

[B1] Bates K. L. Higley M. Letsou A. (2008). Raw mediates antagonism of AP-1 activity in Drosophila. Genetics 178, 1989–2002. 10.1534/genetics.107.086298 18430930 PMC2323791

[B2] Bauer Huang S. L. Saheki Y. VanHoven M. K. Torayama I. Ishihara T. Katsura I.. (2007). Left-right olfactory asymmetry results from antagonistic functions of voltage-activated calcium channels and the Raw repeat protein OLRN-1 in C. elegans. Neural Dev. 2, 24. 10.1186/1749-8104-2-24 17986337 PMC2213652

[B3] Bornstein B. Zahavi E. E. Gelley S. Zoosman M. Yaniv S. P. Fuchs O.. (2015). Developmental axon pruning requires destabilization of cell adhesion by JNK signaling. Neuron 88, 926–940. 10.1016/j.neuron.2015.10.023 26586184

[B4] Brace E. J. Essuman K. Mao X. Palucki J. Sasaki Y. Milbrandt J.. (2022). Distinct developmental and degenerative functions of SARM1 require NAD+ hydrolase activity. PLoS Genet. 18, e1010246. 10.1371/journal.pgen.1010246 35737728 PMC9223315

[B5] Bratkowski M. Xie T. Thayer D. A. Lad S. Mathur P. Yang Y.-S.. (2020). Structural and mechanistic regulation of the pro-degenerative NAD hydrolase SARM1. Cell Rep. 32, 107999. 10.1016/j.celrep.2020.107999 32755591

[B6] Budnik V. Koh Y. H. Guan B. Hartmann B. Hough C. Woods D.. (1996). Regulation of synapse structure and function by the Drosophila tumor suppressor gene dlg. Neuron 17, 627–640. 10.1016/S0896-6273(00)80196-8 8893021 PMC4661176

[B7] Chang Q. Balice-Gordon R. J. (2000). Highwire, rpm-1, and futsch: balancing synaptic growth and stability. Neuron 26, 287–290. 10.1016/S0896-6273(00)81161-7 10839347

[B8] Choi H. M. C. Li Y. Suraj D. Hsia R.-C. Sarkar C. Wu J.. (2022). Autophagy protein ULK1 interacts with and regulates SARM1 during axonal injury. Proc. Natl. Acad. Sci. U. S. A. 119, e2203824119. 10.1073/pnas.2203824119 36375051 PMC9704737

[B9] Chuang C.-F. Bargmann C. I. (2005). A Toll-interleukin 1 repeat protein at the synapse specifies asymmetric odorant receptor expression via ASK1 MAPKKK signaling. Genes Dev. 19, 270–281. 10.1101/gad.1276505 15625192 PMC545892

[B10] Coleman M. P. Höke A. (2020). Programmed axon degeneration: from mouse to mechanism to medicine. Nat. Rev. Neurosci. 21, 183–196. 10.1038/s41583-020-0269-3 32152523 PMC8926152

[B11] Couillault C. Pujol N. Reboul J. Sabatier L. Guichou J.-F. Kohara Y.. (2004). TLR-independent control of innate immunity in Caenorhabditis elegans by the TIR domain adaptor protein TIR-1, an ortholog of human SARM. Nat. Immunol. 5, 488–494. 10.1038/ni1060 15048112

[B12] Czech V. L. O'Connor L. C. Philippon B. Norman E. Byrne A. B. (2023). TIR-1/SARM1 inhibits axon regeneration and promotes axon degeneration. Elife 12, e80856. 10.7554/eLife.80856 37083456 PMC10121217

[B13] Ding C. Wu Y. Dabas H. Hammarlund M. (2022). Activation of the CaMKII-Sarm1-ASK1-p38 MAP kinase pathway protects against axon degeneration caused by loss of mitochondria. Elife 11, e73557. 10.7554/eLife.73557 35285800 PMC8920508

[B14] Eresh S. Riese J. Jackson D. B. Bohmann D. Bienz M. (1997). A CREB-binding site as a target for decapentaplegic signalling during Drosophila endoderm induction. EMBO J. 16, 2014–2022. 10.1093/emboj/16.8.2014 9155027 PMC1169804

[B15] Farley J. E. Burdett T. C. Barria R. Neukomm L. J. Kenna K. P. Landers J. E.. (2018). Transcription factor Pebbled/RREB1 regulates injury-induced axon degeneration. Proc. Natl. Acad. Sci. U. S. A. 115, 1358–1363. 10.1073/pnas.1715837115 29295933 PMC5819420

[B16] Figley M. D. Gu W. Nanson J. D. Shi Y. Sasaki Y. Cunnea K.. (2021). SARM1 is a metabolic sensor activated by an increased NMN/NAD+ ratio to trigger axon degeneration. Neuron 109, 1118–1136.e11. 10.1016/j.neuron.2021.02.009 33657413 PMC8174188

[B17] Foster K. J. Cheesman H. K. Liu P. Peterson N. D. Anderson S. M. Pukkila-Worley R. (2020). Innate immunity in the C. elegans intestine is programmed by a neuronal regulator of AWC olfactory neuron development. Cell Rep. 31, 107478. 10.1016/j.celrep.2020.03.042 32268082 PMC7215899

[B18] Gerdts J. Brace E. J. Sasaki Y. DiAntonio A. Milbrandt J. (2015). SARM1 activation triggers axon degeneration locally via NAD^+^ destruction. Science 348, 453–457. 10.1126/science.1258366 25908823 PMC4513950

[B19] Gerdts J. Summers D. W. Milbrandt J. DiAntonio A. (2016). Axon self-destruction: new links among SARM1, MAPKs, and NAD+ metabolism. Neuron 89, 449–460. 10.1016/j.neuron.2015.12.023 26844829 PMC4742785

[B20] Gerdts J. Summers D. W. Sasaki Y. DiAntonio A. Milbrandt J. (2013). Sarm1-mediated axon degeneration requires both SAM and TIR interactions. J. Neurosci. 33, 13569–13580. 10.1523/JNEUROSCI.1197-13.2013 23946415 PMC3742939

[B21] Gilley J. Coleman M. P. (2010). Endogenous Nmnat2 is an essential survival factor for maintenance of healthy axons. PLoS Biol. 8, e1000300. 10.1371/journal.pbio.1000300 20126265 PMC2811159

[B22] Gilley J. Orsomando G. Nascimento-Ferreira I. Coleman M. P. (2015). Absence of SARM1 rescues development and survival of NMNAT2-deficient axons. Cell Rep. 10, 1974–1981. 10.1016/j.celrep.2015.02.060 25818290 PMC4386025

[B23] Hao Y. Waller T. J. Nye D. M. Li J. Zhang Y. Hume R. I.. (2019). Degeneration of injured axons and dendrites requires restraint of a protective JNK signaling pathway by the transmembrane protein Raw. J. Neurosci., 0016–0019. 10.1523/JNEUROSCI.0016-19.2019 31492772 PMC6807270

[B24] Herrmann K. A. Liu Y. Llobet-Rosell A. Mc Laughlin C. N. Neukomm L. J. Coutinho-Budd J. C.. (2022). Divergent signaling requirements of dSARM in injury-induced degeneration and developmental glial phagocytosis. PLoS Genet. 18, e1010257. 10.1371/journal.pgen.1010257 35737721 PMC9223396

[B25] Inoue A. Sawatari E. Hisamoto N. Kitazono T. Teramoto T. Fujiwara M.. (2013). Forgetting in C. elegans is accelerated by neuronal communication via the TIR-1/JNK-1 pathway. Cell Rep. 3, 808–819. 10.1016/j.celrep.2013.02.019 23523351

[B26] Izadifar A. Courchet J. Virga D. M. Verreet T. Hamilton S. Ayaz D.. (2021). Axon morphogenesis and maintenance require an evolutionary conserved safeguard function of Wnk kinases antagonizing Sarm and Axed. Neuron 109, 2864–2883.e8. 10.1016/j.neuron.2021.07.006 34384519

[B27] Jemc J. C. Milutinovich A. B. Weyers J. J. Takeda Y. Van Doren M. (2012). Raw Functions through JNK signaling and cadherin-based adhesion to regulate Drosophila gonad morphogenesis. Dev. Biol. 367, 114–125. 10.1016/j.ydbio.2012.04.027 22575490 PMC3635074

[B28] Jiang Y. Liu T. Lee C.-H. Chang Q. Yang J. Zhang Z. (2020). The NAD+-mediated self-inhibition mechanism of pro-neurodegenerative SARM1. Nature 588, 658–663. 10.1038/s41586-020-2862-z 33053563

[B29] Lee J. Peng Y. Lin W.-Y. Parrish J. Z. (2015). Coordinate control of terminal dendrite patterning and dynamics by the membrane protein Raw. Development 142, 162–173. 10.1242/dev.113423 25480915 PMC4299136

[B30] Lloyd A. Yancheva N. Wasylyk B. (1991). Transformation suppressor activity of a Jun transcription factor lacking its activation domain. Nature 352, 635–638. 10.1038/352635a0 1907719

[B31] Luong D. Perez L. Jemc J. C. (2018). Identification of raw as a regulator of glial development. PLoS ONE 13, 1–20. 10.1371/journal.pone.0198161 29813126 PMC5973607

[B32] Martín-Blanco E. Gampel A. Ring J. Virdee K. Kirov N. Tolkovsky A. M.. (1998). puckered encodes a phosphatase that mediates a feedback loop regulating JNK activity during dorsal closure in Drosophila. Genes Dev. 12, 557–570. 10.1101/gad.12.4.557 9472024 PMC316530

[B33] McLaughlin C. N. Nechipurenko I. V. Liu N. Broihier H. T. (2016). A Toll receptor-FoxO pathway represses Pavarotti/MKLP1 to promote microtubule dynamics in motoneurons. J. Cell Biol. 214, 459–474. 10.1083/jcb.201601014 27502486 PMC4987293

[B34] McLaughlin C. N. Perry-Richardson J. J. Coutinho-Budd J. C. Broihier H. T. (2019). Dying neurons utilize innate immune signaling to prime glia for phagocytosis during development. Dev. Cell 48, 506–522.e6. 10.1016/j.devcel.2018.12.019 30745142 PMC6394877

[B35] Nimma S. Ve T. Williams S. J. Kobe B. (2017). Towards the structure of the TIR-domain signalosome. Curr. Opin. Struct. Biol. 43, 122–130. 10.1016/j.sbi.2016.12.014 28092811

[B36] Osterloh J. M. Yang J. Rooney T. M. Fox A. N. Adalbert R. Powell E. H.. (2012). dSarm/Sarm1 is required for activation of an injury-induced axon death pathway. Science. 337, 481–484. 10.1126/science.1223899 22678360 PMC5225956

[B37] Ransone L. J. Kerr L. D. Schmitt M. J. Wamsley P. Verma I. M. (1993). The bZIP domains of Fos and Jun mediate a physical association with the TATA box-binding protein. Gene Expr. 3, 37–48. 7685215 PMC6081620

[B38] Ring J. M. Arias A. M. (1993). puckered, a gene involved in position-specific cell differentiation in the dorsal epidermis of the Drosophila larva. Development 119, 251–259. 10.1242/dev.119.Supplement.251 8049480

[B39] Ritzenthaler S. Suzuki E. Chiba A. (2000). Postsynaptic filopodia in muscle cells interact with innervating motoneuron axons. Nat. Neurosci. 3, 1012–1017. 10.1038/79833 11017174

[B40] Rui M. Bu S. Chew L. Y. Wang Q. Yu F. (2020). The membrane protein Raw regulates dendrite pruning via the secretory pathway. Development 147. 10.1242/dev.191155 32928906

[B41] Sanyal S. (2009). Genomic mapping and expression patterns of C380, OK6 and D42 enhancer trap lines in the larval nervous system of Drosophila. Gene Expr. Patterns 9, 371–380. 10.1016/j.gep.2009.01.002 19602393

[B42] Shen C. Vohra M. Zhang P. Mao X. Figley M. D. Zhu J.. (2021). Multiple domain interfaces mediate SARM1 autoinhibition. Proc. Natl. Acad. Sci. USA. 118. 10.1073/pnas.2023151118 33468661 PMC7848697

[B43] Sporny M. Guez-Haddad J. Khazma T. Yaron A. Dessau M. Shkolnisky Y.. (2020). Structural basis for SARM1 inhibition and activation under energetic stress. Elife 9. 10.7554/eLife.62021.sa2 33185189 PMC7688312

[B44] Sporny M. Guez-Haddad J. Lebendiker M. Ulisse V. Volf A. Mim C.. (2019). Structural evidence for an octameric ring arrangement of SARM1. J. Mol. Biol. 431, 3591–3605. 10.1016/j.jmb.2019.06.030 31278906

[B45] Walker L. J. Summers D. W. Sasaki Y. Brace E. J. Milbrandt J. DiAntonio A. (2017). MAPK signaling promotes axonal degeneration by speeding the turnover of the axonal maintenance factor NMNAT2. Elife 6, e22540. 10.7554/eLife.22540 28095293 PMC5241118

[B46] Waller A. V. (1850). Experiments on the section of the glossopharyngeal and hypoglossal nerves of the frog, and observations of the alterations produced thereby in the structure of their primitive fibres. Philos. Trans. R. Soc. Lond. 140, 423–429. 10.1098/rstl.1850.0021 30332247 PMC5929074

[B47] Waller T. J. Collins C. A. (2022). Multifaceted roles of SARM1 in axon degeneration and signaling. Front. Cell. Neurosci. 16, 958900. 10.3389/fncel.2022.958900 36090788 PMC9453223

[B48] Wang Q. Zhang S. Liu T. Wang H. Liu K. Wang Q.. (2018). Sarm1/Myd88-5 regulates neuronal intrinsic immune response to traumatic axonal injuries. Cell Rep. 23, 716–724. 10.1016/j.celrep.2018.03.071 29669278

[B49] Weber U. Paricio N. Mlodzik M. (2000). Jun mediates Frizzled-induced R3/R4 cell fate distinction and planar polarity determination in the Drosophila eye. Development 127, 3619–3629. 10.1242/dev.127.16.3619 10903185

[B50] Xiong X. Collins C. A. (2012). A conditioning lesion protects axons from degeneration via the Wallenda/DLK MAP kinase signaling cascade. J. Neurosci. 32, 610–615. 10.1523/JNEUROSCI.3586-11.2012 22238096 PMC3280217

[B51] Xiong X. Hao Y. Sun K. Li J. Li X. Mishra B.. (2012). The Highwire ubiquitin ligase promotes axonal degeneration by tuning levels of Nmnat protein. PLoS Biol. 10, e1001440. 10.1371/journal.pbio.1001440 23226106 PMC3514318

[B52] Xiong X. Wang X. Ewanek R. Bhat P. Diantonio A. Collins C. A. (2010). Protein turnover of the Wallenda/DLK kinase regulates a retrograde response to axonal injury. J. Cell Biol. 191, 211–223. 10.1083/jcb.201006039 20921142 PMC2953441

[B53] Zhai R. G. Cao Y. Hiesinger P. R. Zhou Y. Mehta S. Q. Schulze K. L.. (2006). Drosophila NMNAT maintains neural integrity independent of its NAD synthesis activity. PLoS Biol. 4, e416. 10.1371/journal.pbio.0040416 17132048 PMC1665629

[B54] Zhou J. Edgar B. A. Boutros M. (2017). ATF3 acts as a rheostat to control JNK signalling during intestinal regeneration. Nat. Commun. 8, 14289. 10.1038/ncomms14289 28272390 PMC5344978

