# Supplementary figures and images for: Opposing roles of Fos, Raw, and SARM1 in the regulation of axonal degeneration and synaptic structure
Source: Front Cell Neurosci. 2023 Nov 30;17:1283995. doi: 10.3389/fncel.2023.1283995 (PMC10719852; doi:10.3389/fncel.2023.1283995)

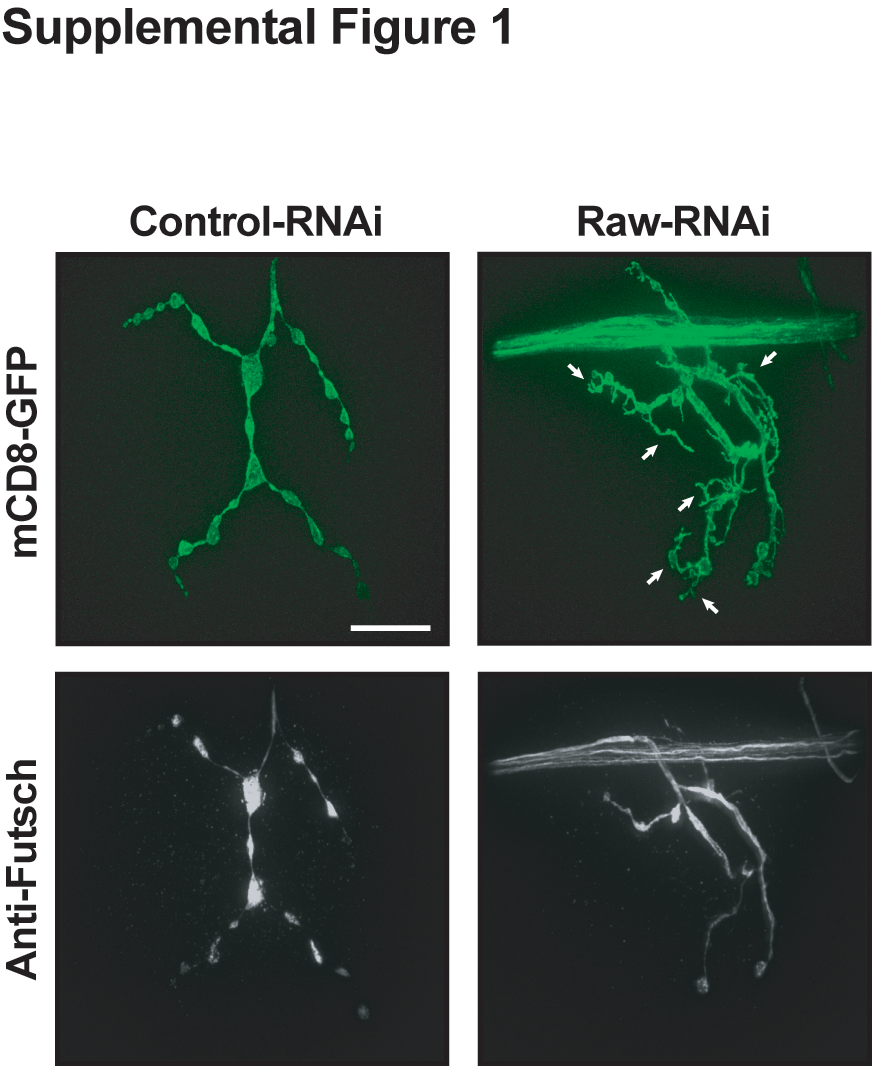

Supplement: Supplementary Figure 1 — Effect of raw knockdown on NMJ structure and Futsch. Representative images of Muscle 4 NMJs from third instar larvae. UAS-mCD8-GFP was expressed in all motoneurons by D42-GAL4. Larvae were co-stained for Futsch (22C10, DHSB). Arrows indicate filopodia-like structures. Dcr2 was expressed in all panels for RNAi efficiency. The scale bar is 20 μm. Error bars show 95% confidence interval. [file Image_1.TIF]

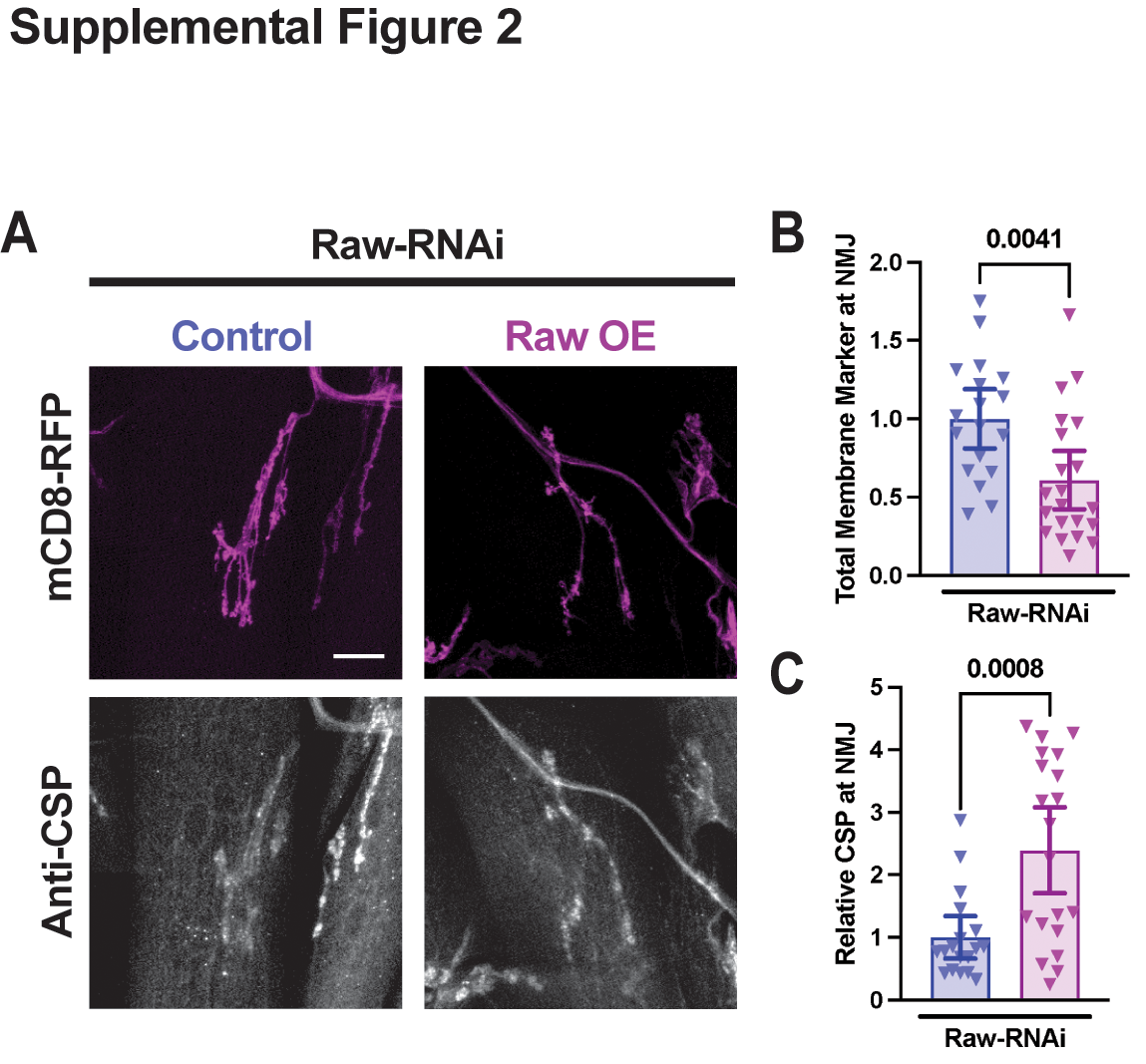

Supplement: Supplementary Figure 2 — Overexpression of Raw rescues NMJ phenotypes of Raw RNAi. Co-expression of UAS-GFP-Raw rescues overgrowth and relative CSP decrease of Raw RNAi. (A) UAS-Raw-RNAi is co-expressed with UAS-mCD8-RFP and UAS-Dcr2 (for RNAi efficiency), together with UAS-GFP-Raw or UAS-Luciferase (control) via the D42-Gal4 driver. The scale bar is 20 μm. The two-tailed unpaired t-test was used for panels (B, C). Error bars show 95% confidence interval. [file Image_2.TIF]

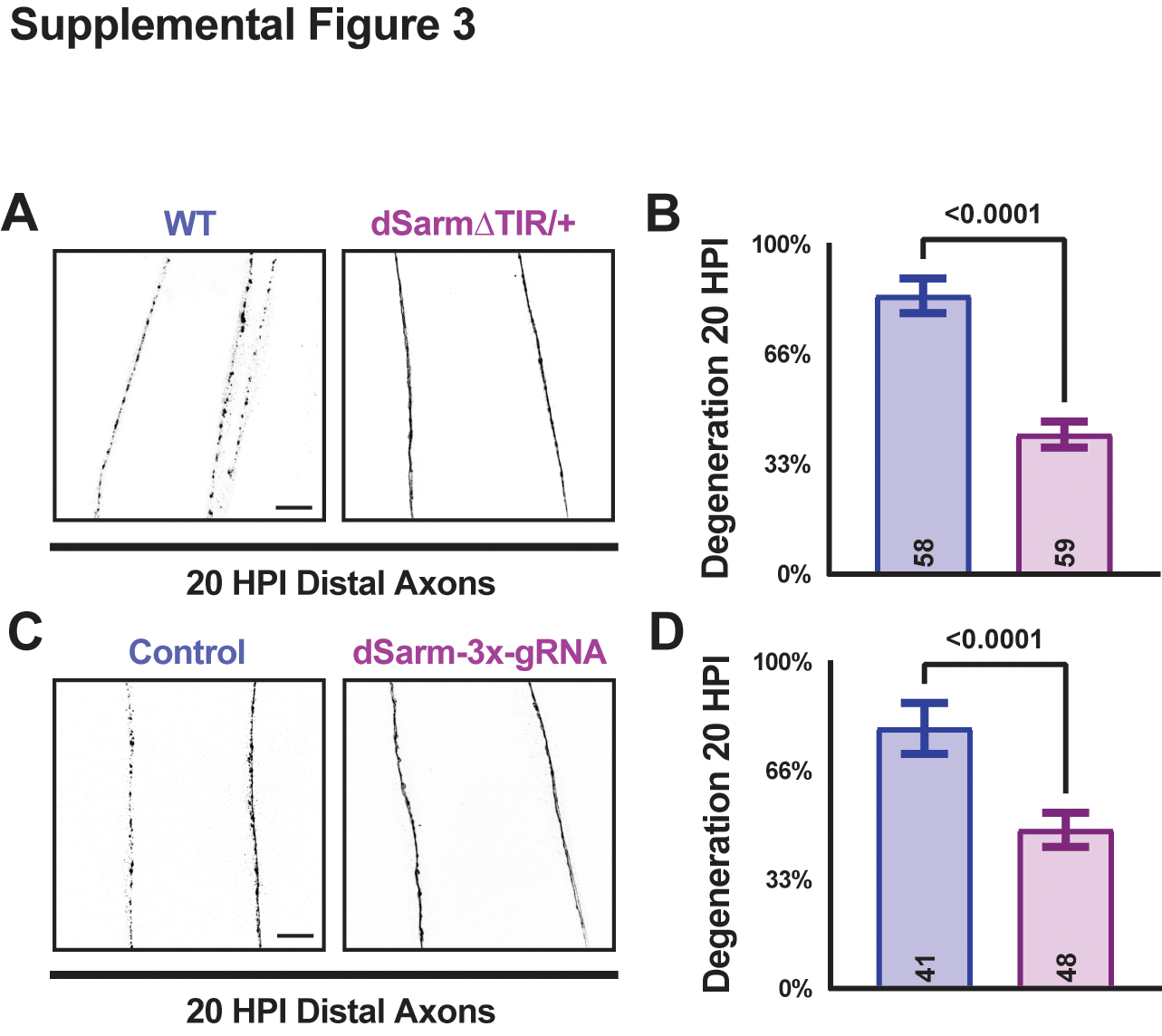

Supplement: Supplementary Figure 3 — Validation of loss-of-function manipulations in dSarm. MNSNc axons are visualized using the mCD8 membrane marker transgene (tagged with either RFP or GFP) driven by m12-Gal4 and assayed for Wallerian degeneration at 20 h postinjury. (A, B) Heterozygous dominant negative mutations of dSarm's catalytic TIR domain, dSarmDeltaTIR/+, were compared to the control (w118) background. (C, D) CRISPR/Cas9 knockdown of dSarm was carried out by co-expression of dSarm-3x-gRNA with UAS-Cas9 using the m12-Gal4 driver. Control flies express a gRNA that targets the QUAS sequence (not present in these flies). The scale bars are 20 μm. The two-tailed unpaired t-test was used for panels (B, D). Error bars show 95% confidence interval. [file Image_3.TIF]
